# Novel use of online optimization in a mathematical model of COVID-19 to guide the relaxation of pandemic mitigation measures

**DOI:** 10.1038/s41598-022-08389-5

**Published:** 2022-03-18

**Authors:** Gianluca Bianchin, Emiliano Dall’Anese, Jorge I. Poveda, David Jacobson, Elizabeth J. Carlton, Andrea G. Buchwald

**Affiliations:** 1 Department of Electrical, Computer, and Energy Engineering, University of Colorado Boulder, Boulder, CO USA; 2 Vanadata, Boulder, CO USA; 3 Department of Environmental and Occupational Health, Colorado School of Public Health, University of Colorado Anschutz, Aurora, CO USA; 4 Department of Biostatistics and Informatics, Colorado School of Public Health, University of Colorado Anschutz, Aurora, CO USA

**Keywords:** Infectious diseases, Infectious diseases, Applied mathematics, Public health

## Abstract

Since early 2020, non-pharmaceutical interventions (NPIs)—implemented at varying levels of severity and based on widely-divergent perspectives of risk tolerance—have been the primary means to control SARS-CoV-2 transmission. This paper aims to identify how risk tolerance and vaccination rates impact the rate at which a population can return to pre-pandemic contact behavior. To this end, we developed a novel mathematical model and we used techniques from feedback control to inform data-driven decision-making. We use this model to identify optimal levels of NPIs across geographical regions in order to guarantee that hospitalizations will not exceed given risk tolerance thresholds. Results are shown for the state of Colorado, United States, and they suggest that: coordination in decision-making across regions is essential to maintain the daily number of hospitalizations below the desired limits; increasing risk tolerance can decrease the number of days required to discontinue NPIs, at the cost of an increased number of deaths; and if vaccination uptake is less than 70%, at most levels of risk tolerance, return to pre-pandemic contact behaviors before the early months of 2022 may newly jeopardize the healthcare system. The sooner we can acquire population-level vaccination of greater than 70%, the sooner we can safely return to pre-pandemic behaviors.

## Introduction

The primary strategy for mitigating the spread of SARS-CoV-2, to date, has relied on the use of non-pharmaceutical interventions (NPIs). Common NPIs include, at various levels of severity, lockdowns, travel restrictions, contact tracing, mask-wearing, and individual behavioral change. These policy-based restrictions and individual behavior changes have had wide-ranging social consequences, including disruptive impacts on economies, and they have severely affected the well-being of families and children due to confinement stress and social disruptions^1,2^. On December 14, 2020, a mass vaccination campaign was initiated in the United States and, as the spread of the SARS-CoV-2 virus slowed, individuals and policy-makers alike are planning a “return to normality”, where policy-makers begin to lift NPIs and individual social behaviors are tentatively resumed. The individual and policy-level decisions to lift NPIs and “return to normal” depend on two main factors: (i) the trajectory of the SARS-CoV-2 states once the NPIs are lifted, and (ii) the risk tolerance policy-makers and individuals are willing to tolerate. Hence, whether or not this return to normal is safe is a question of (i) population-level immunity (are immunity levels—either due to previous infection or vaccination—sufficient to prevent future waves of infection?) and (ii) risk tolerance (what is the largest number of cases, hospitalizations, or deaths individuals and policy-makers altogether are willing to accept?).

Predicting the minimum level of immunity that is needed to remove all NPIs and return to normal behavior remains an open research question, especially in the presence of regional discrepancies in risk tolerance and vaccination rates. Tolerable levels of transmission risk have been a controversial topic throughout the evolution of the SARS-CoV-2 pandemic and could be defined based on number of cases, hospitalizations, or deaths. We chose hospitalizations for this analysis because they are a marker of severe COVID-19 disease and are less lagged than deaths. In these scenarios, we are not pursuing a goal of elimination of cases—which, with evidence of waning immunity and continued global import, is improbable in the near term. Instead, we focus on maintaining the number of cases below a tolerable level of endemic disease burden. Defining the tolerable risk level is outside the scope of this paper—but may be based on infrastructure limits such as hospital capacity, as well as societal values including equity, a desire to minimize the risk of death or severe disease, or a desire to minimize disruption (e.g., school closures). Here we examine a range of possible risk tolerance thresholds as a demonstration.

In this work, we aim to answer the following questions: given the ongoing vaccination campaigns and a range of levels of risk tolerance, when can most NPIs be relaxed (we use the term NPIs to encompass all policy restrictions as well as individual-level behavior change) and life return to normal? How is this return to normal impacted by variations in vaccination rates and risk tolerance? And, how does mobility between regions (counties, states, etc) impact the return to normal, and how to coordinate NPIs across regions?

To answer these questions, we adopt a novel approach for data-driven policy-making using multidisciplinary methods pulling from transmission modeling^3–6^, online optimization of dynamical systems^7–10^, and feedback control^11^. We develop a method that allows us to identify maximum contact levels across geographical regions that guarantee hospitalizations will not exceed a given threshold, while minimizing the economic and social impacts (see “Discussion” section). This aspect is of utmost importance to ensure that future waves of infections do not threaten the stability of public health infrastructures. The operating principles of the proposed method depart from standard approaches in epidemic control that may be based on specific models for the evolution of the epidemic, and may rely on ad hoc decision rules; the proposed controller operates in closed-loop, in the sense that it suggests whether the level of NPIs should increase (or can be decreased) to meet predefined economic objectives and risk tolerance metrics based on the current level of infections and a prediction of the peak of hospitalizations.

The approach is widely applicable and can be implemented at various geographical granularities and to other disease systems. As a test case, we considered the state of Colorado, USA. We calibrated our models using real-world data, and we evaluated the number of days that are required before all NPIs can be relaxed in relation to: (i) risk tolerance; (ii) maximum vaccination uptake; and (iii) daily vaccination rate. We additionally examine the way the introduction of new variants alters these findings. We uncover important aspects related to the timing and coordination of the NPIs in the various regions based on travel patterns.

## Results

### Controlling hospitalizations during pandemic outbreaks

**Figure 1:**
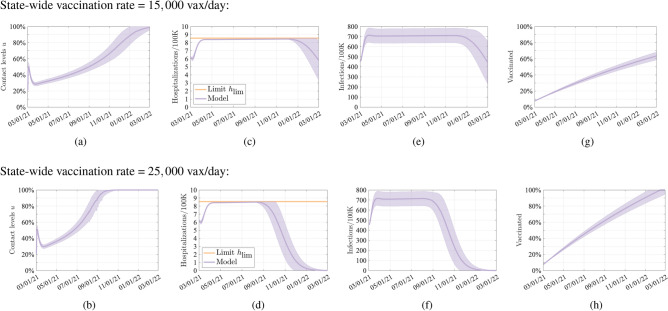
Model behavior when the feedback law is designed to simultaneously maximize contact levels and maintain hospitalizations below the threshold ${h}_{lim }$. (**a,b**) Level of transmission-relevant contacts with respect to pre-pandemic behavior, as selected by the feedback controller. All simulations are conducted by using a single region model that is fitted using data from the state of Colorado, USA (see “Methods” section). Results are averaged over 10,000 simulations with parameters sampled using a Latin Hypercube technique within 15% of their nominal values. Continuous line shows mean of the trajectory and shaded area show 99.73% confidence intervals. This figure shows an ideal situation where vaccination uptake can reach a level of 100%.

**Figure 2:**
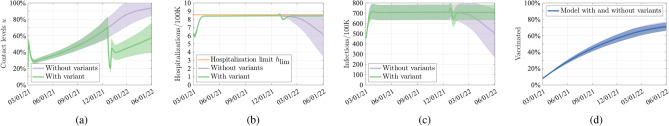
Model and controller behavior without variant (purple and blue lines) and with a more infectious variant (green and blue lines). The effect of the variant is modeled by doubling the transmission rate on 12/21/21. The feedback law is designed to simultaneously maximize contact levels and maintain hospitalizations below the threshold ${h}_{lim }$. All simulations are conducted by using a single-compartment model that is fitted using data from the state of Colorado, USA (see “Methods” section). Results are averaged over 10,000 simulations with parameters sampled using a Latin Hypercube technique within $15\%$ of their nominal values. Continuous line shows mean of the trajectory and shaded area show 99.73% confidence intervals.

We aim to address the following question: “What is the least restrictive level of NPIs that guarantees that the number of hospitalized individuals on each day do not exceed a pre-specified limit and simultaneously accounts for the economic implications of the NPIs?”. The hospitalization limit models the level of risk tolerance in a population or the level of stress tolerated by the healthcare system in a given region. We answer this question by formulating a constrained optimization problem, which uses a compartmental model of epidemic transmission to predict the epidemic state (see “Methods” section). The intensity of NPIs is represented by a parameter $u \in [0,1]$ that describes the level of permitted transmission-relevant contacts, where $u=0$ corresponds to zero contacts (full lockdown, $0\%$ contact levels) and $u=1$ corresponds to pre-pandemic contact levels (zero NPIs, $100\%$ contact levels) (see Figs. 1a,b,  2a). An optimization problem is used to derive a feedback law that uses the instantaneous epidemic state to systematically select a level of transmission-relevant contacts that balance between the economic impact of the imposed restrictions and the number of infectious individuals, while simultaneously guaranteeing that the number of daily hospitalizations does not exceed the specified hospitalization limit (Fig. 1c,d), denoted by ${h}_{lim }$. Here, ${h}_{lim }$ models the maximum allowable number of hospitalized individuals on each day. The parameters of our model are fitted to official data from the state of Colorado, USA^12,13^. Given the emergence of new, highly transmissible variants, we conducted a sensitivity analysis to examine the impact of these variants on our results (Fig. 2), simulated by assuming doubling transmission rates, akin to what has been seen with the Delta and Omicron variants. Note that in the absence of new variants, our model predicts that *u* will gradually return to 100%. If we assume the repeated introduction of new variants, *u* has to repeatedly reset to a lower level, delaying its approach to 100%. This would result in temporary states where returns to high contact levels is intermittently safe, but also suggesting contact levels have to be repeatedly reduced, as has been seen in actuality.

### When can we safely return to normal?

To address this question, we used the feedback optimization framework to determine the highest allowable level of contacts (on each day) that ensures that the daily number of hospitalized individuals does not exceed (and remains close to) the pre-specified limit ${h}_{lim }$. In a single simulation (Fig. 1) it can be seen that as the fraction of vaccinated individuals in the population increases, the allowable contact levels selected by the feedback law can also gradually increase while ensuring that the number of hospitalizations remains below the predefined limit; the infection rate is similarly constrained. Accordingly, our framework allows us to characterize a lower bound on the number of days required before a full return to normality can safely occur.

**Figure 3:**
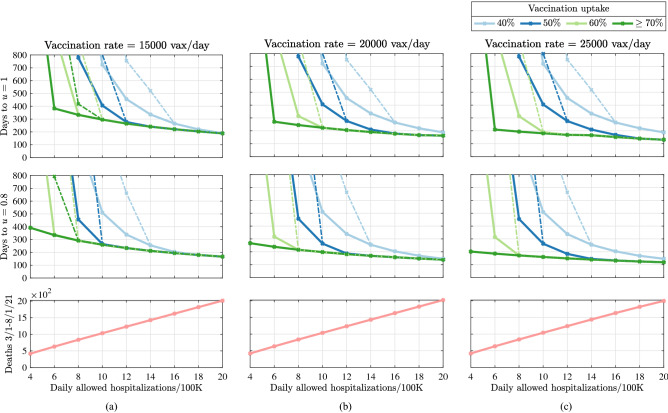
Number of days needed before a return to normal can be implemented without exceeding predefined hospitalization limits. The number of days is counted beginning 03/01/21. Continuous lines illustrate counts when models are not affected by variants. Dashed lines illustrate counts when models are affected by a variant whose effect is to double the transmission rate on 12/21/21. (Top row) Number of days to $u = 1$. (Center row:) Number of days to $u=0.8$. (Bottom row) Estimated number of deaths between 03/01/21, and 08/01/2021. All simulations are conducted by using a single-compartment model fitted using data from the state of Colorado, USA (see “Methods” section). Any vaccination uptake of $70\%$ or larger yields an identical (dark green) curve.

Return to normality refers to a condition where all NPIs can be repealed and the societal behavior can return to pre-pandemic contact levels (i.e. $u = 1$). We found that the parameter that most consistently impacted the number of days to normality is the vaccination uptake in the population (see Fig. 3 where the number of days to normality is counted beginning 03/01/21). This finding follows from three main observations. First, our results show that any vaccination uptake of 50% or less will require more than 2 years (730 days) to return to normal behavior for any of the examined vaccination rates if we expect to keep the number of hospitalizations below 8 individuals/day per 100 K inhabitants. Second, the number of days to normality reduces by a factor of at least 2.5 as the vaccination uptake is increased from 50 to 60% (for instance, with a vaccination rate of 25,000 vax/day and hospitalization limit ${h}_{lim } = 8$, the number of days to $u=1$ decreases from 777 to 314 as the vaccination uptake is varied from 50 to 60%). Third, our results suggest that vaccination uptakes larger than 70% will not decrease the time before all NPIs can be safely lifted. This is likely a factor of the current level of infection-derived immunity in the population, such that 70% vaccine uptake is sufficient to reach a threshold where infections decline, regardless of contact levels. A second parameter that consistently affects the time to normality is the level of risk tolerance ${h}_{lim }$, i.e., the number of hospitalizations tolerated during the outbreak. Precisely, allowing more hospitalizations to occur (increasing ${h}_{lim }$) reduces the number of days to normality. Decreased risk tolerance leads to a lower infection rate and decreases the rate of naturally-acquired immunity, thus increasing the time required for a return to normality. For instance, with a vaccination rate of 15,000 individuals/day, when vaccination uptake is at least 70%, the number of days to $u=1$ reduces from over 1 year (383 days) to about 6 months (189 days) when the hospitalization limit is increased from 6 to 20 individuals/day. Unfortunately, although a higher hospitalization limit reduces the time to normality thanks to naturally-acquired immunity, it also results in a higher number of deaths (Fig. 3 bottom panel). Our simulation outcomes also suggest that the vaccination uptake does not affect the number of deaths in the considered time interval (this fact emerges because the number of deaths is counted until August 1, which occurs before the vaccination uptake threshold is reached).

Our results suggest that to return to normal behavior (pre-pandemic contact rates) on 10/01/21 (153 days beginning 03/01/21), there is likely to be stress on the healthcare system (i.e. the number of hospitalizations will exceed the pre-defined limit), the level of which will depend on vaccine uptake. If vaccine uptake is as low as 40%, we may not remain below 20 hospitalizations/day per 100 K inhabitants. However, with a vaccination uptake of 60% we could return to normal and keep hospitalizations below 16 hospitalizations/day per 100 K inhabitants.

In practice, we expect that contact behaviors will vary widely across individuals and that, even when all government policies will be repealed, many individuals will likely continue to practice NPIs, such as mask-wearing and social distancing. Thus, although $u = 1$ may be an unlikely scenario in the near future, individuals may quickly resume a behavior of “almost normality”, where contacts are restored to 80% of pre-pandemic levels (i.e., $u = 0.8$).

Following that, in order to safely return to $u = 0.8$ on 10/01/21, if vaccine uptake is as low as 50%, we may not remain below 14 hospitalizations/day per 100 K inhabitants. However, with vaccine uptake of 70% we could return to normal and keep hospitalizations below 12 hospitalizations/day per 100 K inhabitants (see Fig. 3 center row).

### Effects of regional heterogeneities and mobility patterns

**Figure 4:**
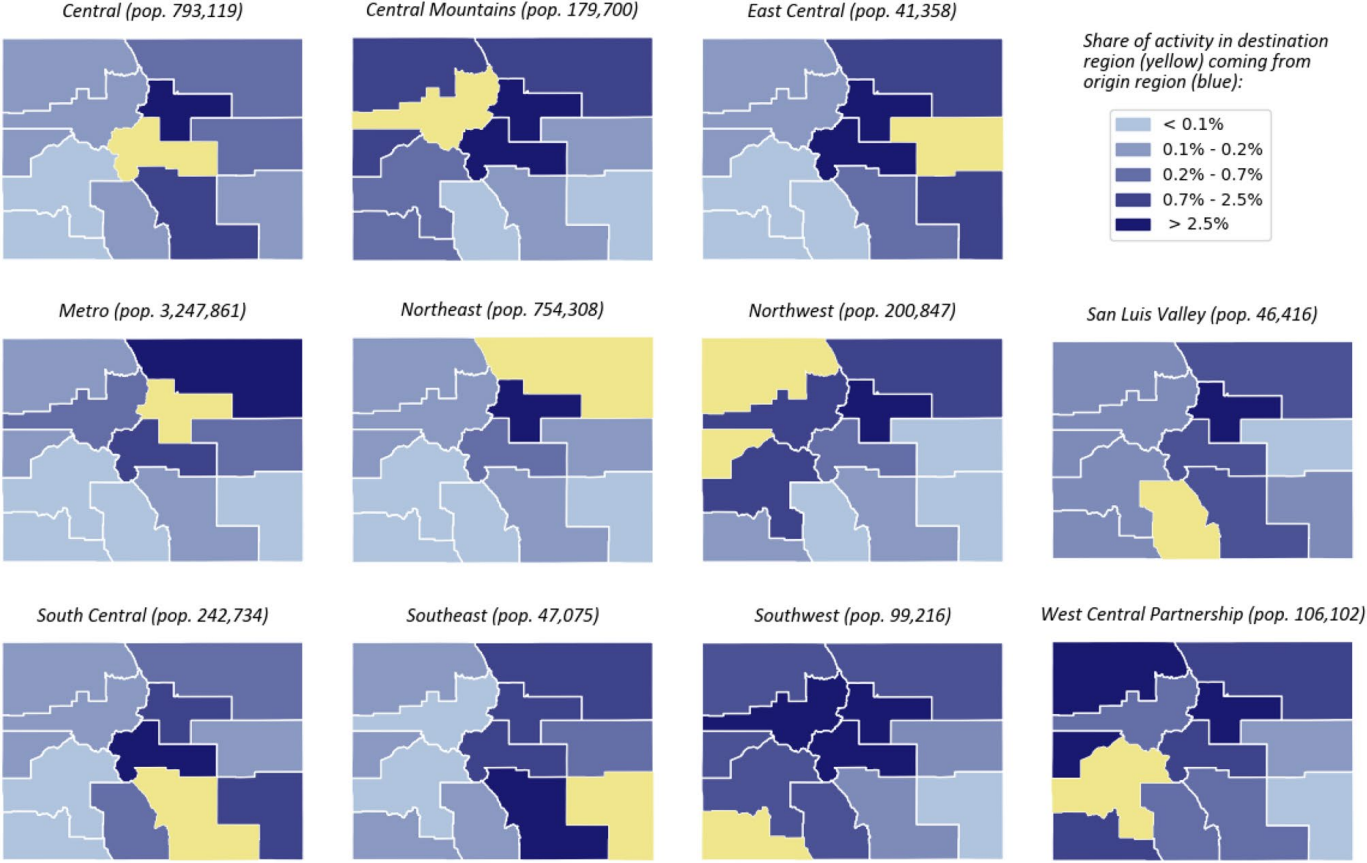
Regional connectivity patterns between the 11 Local Public Health Agency regions in Colorado, USA. Each panel illustrates the intensity of contact between residents of the yellow region and individuals traveling from the blue regions. Total travel volume is averaged over the time period 01/01/20–12/31/20. Data obtained from Safegraph (see Data availability).

Heterogeneities in policies and behavioral responses to an ongoing epidemic between regions motivate the use of higher-resolution models and control techniques that can adequately capture this diversity. In this context, a crucial open question concerns how local authorities can identify region-dependent levels of NPIs that guarantee that local hospitalization limits are met, and to what extent inter-regional decision-making can be coordinated to achieve this objective. To address these questions, we generalized the transmission model and feedback control framework to a network setting (see “Methods” section), and we used publicly-available mobility data from cell phone usage to estimate inter-regional couplings (Fig. 4 illustrates regional connectivity patterns for the state of Colorado, USA. Note that the Metro region comprises the majority of the state’s population and contributes to a large fraction of economic activity statewide. The model is organized into eleven regions, each describing a Local Public Health Agency (LPHA) region in Colorado, USA^13^, and transmission levels are fitted to regional hospitalization data from the period 01/01/21–02/28/21.

**Figure 5:**
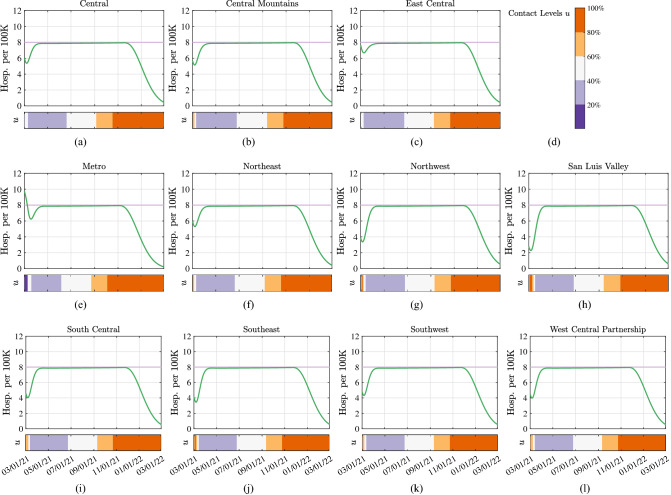
Hospitalizations and controller level over time when a group of regional controllers are used to guarantee that pre-specified region-dependent hospitalization limits are not violated. Each of the 11 panels shows the evolution in a different LPHA region (see Fig. 4 for an illustration of the connectivity graph). Simulation are conducted with a state-wide vaccination rate of 20,000 vax/day, to a maximum vaccine update of 70%. Solid green lines illustrate the evolution of the hospitalized state, light purple lines show the pre-specified hospitalization limit. Heat maps illustrate the required level of NPIs $u_i$, as determined by the controller.

Local levels of NPIs in each region *i* are represented by a parameter $u_i \in [0,1]$, describing the level of permitted transmission-relevant contacts (or NPIs) in the region. By using the feedback-optimizing control law (see “Methods” section), we derived region-dependent daily levels of NPIs that guarantee that the number of hospitalized individuals in each region *i* does not exceed a region-dependent hospitalization limit ${h}_{{lim ,i}}$. Figure 5 illustrates the number of hospitalized individuals in the time-interval 03/01/21–03/01/22, together with the contact levels $u_i$, as selected by the controller. By comparing the simulation outcomes across the various regions, our model and control methods suggest that heterogeneities among the regions can be exploited by the feedback controller, which is capable of meeting hospitalization limits in all regions by devising levels of NPIs that are region-specific. After the initial phase, uniform levels of NPIs among the regions can be used provided that all regions have homogeneous hospitalization limits. Together, our results indicate that regional heterogeneities can be used by the feedback controller, especially when the epidemic state varies widely across the regions. Note that control levels and epidemic curves varied drastically between regions throughout the pandemic, indicating, among other heterogeneities, the difficulty in agreeing upon tolerable risk levels in a heterogeneous region.

### The value of coordination

**Figure 6:**
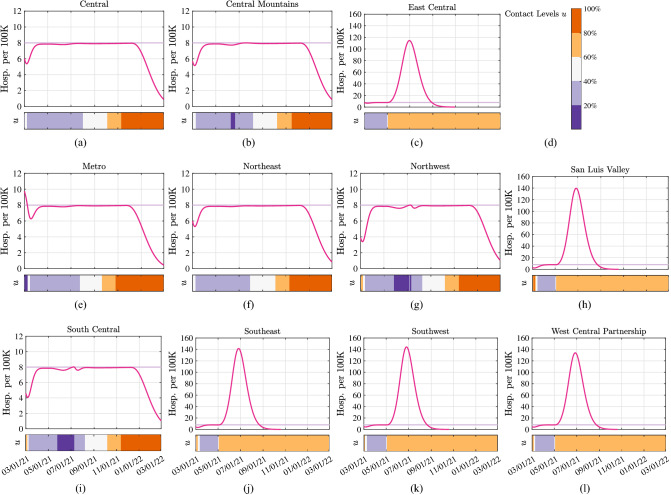
Hospitalizations and controller level over time when regions with a population of 150,000 people or less (i.e., East Central, San Luis Valley, Southeast, Southwest, West Central Partnership) drop all NPIs on 05/01/21. Simulation conducted with vaccination rate $y=20,000$ vax/day. Each of the 11 panels shows the evolution in time in a different LPHA region (see Fig. 4 for an illustration of the connectivity graph). Solid magenta lines illustrate the evolution of the hospitalized state, light purple lines show the pre-specified hospitalization limit. Heat maps illustrate the required level of NPIs $u_i$, as determined by the controller. Note that y-scale differs between panels.

Figure 6 considers a scenario where regions with a population of 150,000 people or less (i.e., East Central, San Luis Valley, Southeast, Southwest, and the West Central Partnership regions) drop most NPIs as of 05/01/21, returning to 80% contact levels ($u = 0.8$). On this date, the average fraction of fully vaccinated individuals across the state is 21.29%. As illustrated by the simulation, such policy will result in a substantial violation of the hospitalization limit in all of the five regions that drop the NPIs. Not surprisingly, the regions that decrease NPIs on 05/01/21, are also the ones that are affected by the highest number of hospitalizations, with peaks of over 140 hospitalizations/day per 100 K inhabitants around 07/01/21. Interestingly, our results suggest that in this case three highly-populated regions (Central Mountains, Northwest, and South Central) are required to decrease the fraction of contact-relevant interactions to below 20% in order to not exceed the hospitalization limit of 8 individuals/day per 100 K inhabitants. This suggests that high-population regions are at high risk of outbreaks as a result of low levels of control in rural regions. Note that without increasing control in, for instance, the Northwest region, in response to low control levels in surrounding rural areas, the Northwest region would be at risk of large increases in hospitalizations. Together, our results indicate that regional heterogeneities can be exploited by the feedback controller to alleviate the necessary severity of NPIs to stay below hospitalization limits in interconnected regions, however, uncoordinated changes of NPIs in some of the regions will in general impact the level of NPIs imposed in all the remaining regions.

We conclude by noting that, although our models are fitted to hospitalization data from the state of Colorado, and the simulations in Figs. 5 and 6 are performed for this case study, the proposed control framework and feedback control law are applicable to any geographical region and can be implemented at a different geographical granularity.

## Discussion

Vaccinations effectively help to contain the spread of an epidemic by quickly developing immunity in vaccinated individuals, without causing severe illness. Vaccinated individuals are no longer susceptible to infection and, as the proportion of the population that is susceptible decreases, the less likely any social contact will lead to a viral transmission. With sufficient vaccination rates and high vaccine uptake, NPIs can be gradually lifted and social interactions can partially resume in the interest of re-establishing economic and social activities. Despite optimism over widespread vaccination, a safe return to pre-COVID contact behaviors (corresponding to zero NPIs), may still be a long way away, dependent on the number of SARS-CoV-2 infections and consequent severe COVID-19 cases we are willing to tolerate. Additionally, the likely continued emergence of new variants may mean sustained periods of “normal” behavior leads to repeated spikes in hospitalizations and deaths. Indeed, if vaccine uptake remains low, policymakers should face the possibility of having to either tolerate a high level of NPIs, or a high number of severe COVID-19 hospitalizations in the foreseeable future. Alternately, higher vaccination rates, if they can be achieved, could lead to a quicker return to normal activities.

We examined the conditions under which all NPIs can be safely reverted and individuals can resume pre-pandemic contact behaviors. We successfully showed that the adopted control method can be used to identify necessary increases or decreases in NPIs based on the level of community risk tolerance or how severe the hospitalization burden can be tolerated before governments or individuals will act to impose restrictions or change their behaviors.

Control of epidemics is a research area with extensive prior works (see, e.g.^14–20^ and pertinent references therein), which include a variety of methodologies that build on model predictive control, model-based optimal control, periodic lock-downs, etc, or simply heuristics driven by conventional wisdom. Here, we take a novel approach based on online optimization methods for dynamical systems^8,9,21^. Online optimization provides powerful tools to simultaneously control a dynamical system and steer it to an optimal state configuration, where optimality is quantified according to a pre-specified cost function and constraints that embed economic and risk tolerance metrics. Model uncertainties constitute the major complexity of the control task at hand and call for the development of novel control tools that can determine how NPIs can be updated over time under limited model knowledge. The online optimization methods utilized in this work leverages feedback from the system to adaptively update the control variables in the face of possible model uncertainties and externalities. Here, the evolution of the pandemic is controlled based on objectives embedded in the optimization problems and by relying on the current number of infectious individuals and a prediction of the function that maps contact levels into the number of hospitalizations. The latter can be obtained from data generated by using a transmission model (the approach taken in this paper) or using machine learning tools. The setting investigated here also calls for new theoretical endeavors to uncover the stability properties of networked nonlinear dynamical systems modeling the progression of the epidemics and data-driven controllers, especially when the underlying transmission model accounts for the loss of immunity.

In this work, we use a limit on hospitalizations, representing plausible risk tolerance thresholds and stress of the healthcare system, to examine the role of vaccination rate and vaccine uptake on minimum necessary levels of contact. If we assume a low risk-tolerance of no more than 8 individuals hospitalized/day per 100K inhabitants, our results suggest the intriguing possibility that the number of days to normality decreased by a factor of two as vaccination uptake in the population is increased from 50 to 60%. While increasing vaccination rates will lead to a decreased “time to normal”, under conservative levels of risk tolerance, safe return to normal may not occur until early 2022. Allowing for increased burden of hospitalizations decreases the time to a safe return to normal behavior, but with serious consequences in the form of increased morbidity and mortality, even under scenarios with high vaccination rates. Vaccine uptake is a key factor for the return to normal. Figure 3 illustrates the number of days before all NPIs can be lifted in relation to different levels of hospitalization limits, daily vaccination rate, and maximum vaccine uptake. Our results suggest that when the hospitalization limit is maintained below 10 individuals/day every 100 K inhabitants, at least 300 days are necessary to lift all NPIs (given a vaccination rate of 15,000 vaccines/day). When the vaccination uptake is as low as $40\%$, this prediction increases to about 700 days. Figure 3c illustrates the cumulative number of deaths from 03/01/21 for different levels of allowed hospitalizations and vaccination rates. Our results suggest that the number of deaths grows linearly with the number of allowed hospitalizations, independent of the rate or uptake of vaccinations. This behavior is due to model assumptions, such that the number of deaths is proportional to the number of infections and hospitalized individuals, which are maintained constant over time by the control method. In practice, this assumption does not hold as vaccinations decrease the death rate among hospitalized individuals proportional to the high-risk population vaccinated. Likewise, new variants have been less virulent and may continue to have lower hospitalization rates, but this model does not account for this heterogeneity.

Regional heterogeneity complicates this picture. Even when only low-population regions with relatively low contact rates may begin to return to relative normality far more quickly, mobility across regions plays a key role. Due to people moving and interacting across regions, removing NPIs too quickly even in regions of low population density can still lead to dire consequences in nearby high-density regions. In our interconnected world, these findings can be generalized to both smaller and greater spatial scales; the application of our method can unveil important intrinsic dependencies that should be fully taken into account to effectively control the spread of the infection.

We acknowledge that our findings come with some relevant limitations. First, the outcomes are dependent on numerous assumptions about baseline transmission rate, probability of hospitalization, and parameter values estimated from previous modeling studies that are specific for the state of Colorado and that may impact our results. Second, we chose not to account for age or the differences between asymptomatic and symptomatic transmission for simplicity. Third, despite accounting for regional heterogeneity in contact rates and baseline transmission, superspreader events and smaller non-homogeneous spatial units play a large role at this stage in the pandemic. Compounding this, vaccine distribution is occurring in a manner that reinforces pre-existing health disparities, due to issues of both access and hesitancy^22^. This creates pockets of high-risk unvaccinated populations, which are sufficient to sustain transmission, even with high vaccination rates overall. Our model cannot account for this type of clustering of behavior or risk, which is important in understanding the probability of achieving sufficiently low levels of SARS-CoV-2 transmission. Fourth, while we ran a single sensitivity analysis to see the impact of increasing transmission rates of new variants, we do not account for the recent introduction and proliferation of numerous variant strains which have the potential to substantially alter transmission dynamics, hospitalization rates, and vaccine efficacy^23^; future work will better account for variants and include them in the proposed methodology. Over time, current vaccines may be less effective at preventing infection due to new circulating variants, preventing the attainment of herd immunity even with high rates of vaccination uptake. Similarly, for this study, we assumed a duration of vaccine effectiveness of two years. Since beginning this work, we have learned the duration of immunity against infection is relatively shorter, however, vaccines do appear to provide long-lasting immunity against SARS-CoV-2 related hospitalization and death, particularly if regular booster shots are provided. If new variants lead to a shorter duration of vaccine-derived immunity, given feasible vaccination rates, complete relaxation of NPIs might never be attainable^24^. When better data on the duration of immunity become available, the model utilized here can be modified accordingly. Fifth, we also acknowledge that it is challenging to translate our control variable into precise NPI policies, such as mask mandates, school closures, business capacity limits, and especially personal decisions. However, ongoing research is evaluating the effects of several (past) interventions on the reproduction number^25,26^, and thus we envision that desired levels of NPIS can be modeled with increasing accuracy shortly. Finally, recently emerged SARS-CoV-2 variants have had higher transmission rates than estimated early in the pandemic. Higher transmission rates will require higher levels of population-level immunity to prevent future outbreaks, and the 70% threshold estimated here may be a severe underestimate of necessary vaccination rates.

Our findings are in agreement with previous modeling studies which have stressed the need to maintain current levels of NPIs and decreased contact for the near future, even in the context of current vaccination strategies^6,27,28^. Several recent studies have also questioned whether herd immunity through vaccination is achievable at all, given the current vaccines available and the high prevalence of vaccine hesitancy^29^. Given these factors, the possibility has been raised that SARS-CoV-2 will become an endemic virus circulating regularly in the population^30^ and our concept of a “return to normal” will have to be reframed. Although herd immunity may not be achievable, our findings suggest that a sufficiently-high vaccination uptake may be sufficient to return to pre-pandemic social behavior. Currently (11/05/21), in the state of Colorado, transmission is rising rapidly, with approximately 1300 individuals currently hospitalized. Vaccination rates are dropping rapidly and whether or not we can reach 70% vaccine uptake is uncertain.

## Methods

The evolution of the epidemic is modeled by using a Susceptible-Exposed-Infectious-Hospitalized-Recovered-Vaccinated-Susceptible (SEIHRVS) compartmental model. We begin by illustrating the single-region model, we then extend the model to account for regional heterogeneities, and lastly, we illustrate the control method.

### Single-region modeling

**Figure 7:**
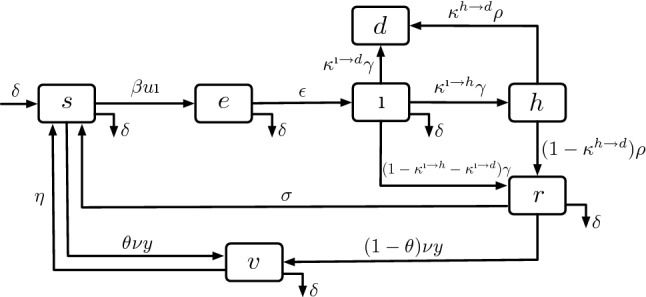
Block diagram of the compartmental model adopted to generate data. The illustrated model is used to describe a single-region. Model equations and extensions to the multi-region model are discussed in “Methods” section.

We utilize a variation of the Susceptible-Exposed-Infectious-Recovered (SEIR) model^31^ that accounts for hospitalizations, vaccinations, and loss of immunity. In particular, we consider a transmission model with states: Susceptible (*s*), Exposed (*e*) Infectious ($\imath$), Hospitalized (*h*), Recovered (*r*), Vaccinated (*v*), and Deceased (*d*). Infectious individuals can infect susceptible ones with a transmission rate $\beta > 0$. To model NPIs, we let $u \in [0,1]$ be a scalar parameter that specifies the level of permitted social activity (or contact levels) within the region. The special case $u = 0$ models a full lock-down, while $u = 1$ corresponds to “zero” NPIs (and hence, a return to pre-pandemic contact levels). Noticing that different levels of NPIs result in different transmission-relevant contact levels, the overall model of epidemic transmission is given by the following differential equations:

1 $$\begin{aligned} \dot{s}&= - \beta u s \imath - \theta \nu y - \delta s + \delta + \sigma r + \eta v,\nonumber \\ \dot{e}&= - \epsilon e - \delta e + \beta u s \imath ,\nonumber \\ \dot{\imath }&= - \gamma \imath - \delta \imath + \epsilon e, \nonumber \\ \dot{h}&= - \rho h + \kappa ^{\imath \rightarrow h} \gamma \imath ,\nonumber \\ \dot{r}&= -\sigma r - \delta r - (1-\theta ) \nu y + (1- \kappa ^{\imath \rightarrow h} - \kappa ^{\imath \rightarrow d})\gamma \imath + (1- \kappa ^{h \rightarrow d})\rho h,\nonumber \\ \dot{v}&= - \eta v - \delta v + \nu y \nonumber ,\\ \dot{d}&= \kappa ^{\imath \rightarrow d} \gamma \imath + \kappa ^{h \rightarrow d} \rho h, \end{aligned}$$

where we use $\dot{x}:=\frac{d}{dt}x(t)$ to denote the time-derivative of a scalar-valued variable *x*(*t*) that is a function of time *t*. In (1), whenever $u \in [0,1)$, the effective transmission rate is reduced from $\beta$ to $\beta u$ according to the imposed NPIs. Individuals become infectious after an incubation period $1/\epsilon >0$, and they recover at a rate $\gamma > 0$. After being infectious, a fraction of individuals $\kappa ^{\imath \rightarrow d} \in [0,1]$ dies, and a fraction $\kappa ^{\imath \rightarrow h} \in [0,1]$ is hospitalized. The fraction $1-\kappa ^{\imath \rightarrow d}- \kappa ^{\imath \rightarrow h}$ quantifies the individuals who recover without hospitalization. Hospitalized individuals recover at rate $\rho >0$. After being hospitalized, a fraction $\kappa ^{h \rightarrow d} \in [0,1]$ of individuals die, while $1-\kappa ^{h \rightarrow d}$ recover. Recovered individuals lose immunity at a rate $\sigma >0$, thus returning in the susceptible compartment. We denote by $y>0$ the vaccination rate and by $\nu \in [0,1]$ the vaccination efficacy. Individuals are vaccinated regardless of their prior infection history and we let $\theta \in [0,1]$ be the fraction of vaccines that is administered to individuals in the *s* compartment, while $(1-\theta )$ vaccines are administered to individuals in the *r* compartment. Finally, $\delta >0$ describes the population birth/death rate. The compartmental model corresponding to the differential equations (1) is illustrated in Fig. 7.

### Multi-region and mobility modeling

We consider a model of disease transmission that is organized into a group of geographical subregions, where individuals make short-term (e.g. daily) inter-regional movements or transits. The assumption that transits are short-term models scenarios where individuals return to the corresponding region of residence immediately after, eventually, being infected. Figure 4 illustrates the partitioning of the state of Colorado according to Local Public Health Agency (LPHA) regions, and illustrates the flow of mobility between regions.

To model contact-relevant interactions among residents of different regions, we adopt a graph $\mathscr {G} = (\mathscr {V}, \mathscr {E})$ where $\mathscr {V} = \{1,\dots ,N\}$ denotes the set of nodes (regions), and $\mathscr {E} \subseteq \mathscr {V} \times \mathscr {V}$ denotes the set of edges (links between regions). We model the coupling between regions by assuming that a fraction $a_{ij} \in [0,1]$ of residents of region *j* travel to region *i* and interact with its residents, with $\sum _{j} a_{ij}=1$ Equivalently, $a_{ij}$ is a normalized parameter that models the intensity of infections due to interactions between infected individuals from region *j* and susceptible individuals from the region *i*. To model the epidemic spread in the regions, we assume that each individual that is a resident of region *i* is categorized into one of the seven compartments $s_i$, $e_i$, $\imath _i$, $h_i$, $r_i$, $v_i$, $d_i$ (where the subscript *i* denotes the index of the region). Similarly to (1), each state of (2) represents the fraction of individuals in the corresponding compartment, with $s_i+e_i+\imath _i+r_i+h_i+v_i+d_i=1$ at all times. The extension of (1) to a multi-region setting is given by:

2 $$\begin{aligned} \dot{s}_i&= - \sum _{j=1}^N \beta u_i a_{ij} s_i \imath _j - \theta _i \nu _i y_i - \delta _i s_i + \delta _i + \sigma _i r_i + \eta _i v_i,\nonumber \\ \dot{e}_i&= - \epsilon _i e_i - \delta _i e_i + \sum _{j=1}^N \beta u_i a_{ij} s_i \imath _j,\nonumber \\ \dot{\imath }_i&= - \gamma _i \imath _i - \delta _i \imath _i + \epsilon _i e_i, \nonumber \\ \dot{h}_i&= - \rho _i h_i + \kappa ^{\imath \rightarrow h}_i \gamma _i \imath _i,\nonumber \\ \dot{r}_i&= -\sigma _i r_i - \delta _i r_i - (1-\theta _i ) \nu _i y_i + (1- \kappa ^{\imath \rightarrow h}_i - \kappa ^{\imath \rightarrow d}_i) \gamma _i \imath _i + (1- \kappa ^{h \rightarrow d}_i)\rho _i h_i,\nonumber \\ \dot{v}_i&= - \eta _i v_i - \delta _i v_i + \nu _i y_i \nonumber ,\\ \dot{d}_i&= \kappa ^{\imath \rightarrow d}_i \gamma _i \imath _i + \kappa ^{h \rightarrow d}_i \rho _i h_i. \end{aligned}$$

We note that (2) allows for different levels of NPIs across the regions, where the variable $u_i \in [0,1]$ describes the permitted level of transmission-relevant contacts within region *i*. This model is motivated by recent works that found the regional progress of influenza much more correlated with the movement of individuals rather than geographic distances^32^. For these reasons, network models are widely used in the literature to take into account spatial propagation effects^31,33–37^.

### Defining a metric for control

For the purpose of this study, we utilize a single control variable per region to encompass all policy measures and behavioral changes in a region that decrease transmission of SARS-CoV-2. This variable captures the impact of several policy measures: including mask mandates, school closures, business capacity limits, as well as personal decisions such as hand-washing, mask-wearing, and moving socialization outside that otherwise would have occurred inside. At present, it is practically intractable to disentangle the specific impact that individual interventions have, as multiple complex interventions are introduced simultaneously and the population is reacting continuously to changing risk perception influenced by divergent policy and messaging at the local, state, and federal level^38^. While NPIs implemented by policy-makers are important, a large portion of transmission reducing behaviors is a result of individual-level risk assessment and behaviors, in response to perceived community transmission^39^. Thus, even as policymakers begin to relax NPIs at the state and regional levels, individuals will continue to make decisions based on their perceptions of risk, which are directly impacted by hospitalization and infection levels in the community. As a result of these effects, we recognize that an actual implementation of various NPIs may have a high variance. For example, even if all policy measures are lifted, as prevalence remains high, it might be unlikely that some individuals will return to pre-pandemic contact behavior. Likewise, even at the height of restrictions, when stay at home orders were in place, it may not be possible to control transmission entirely given the necessity of ongoing essential work, grocery shopping, etc (therefore, *u* can approach 0, but cannot be set to $u = 0$ in practice), or because of possible violations of restrictions.

### Feedback optimization theory for NPI

For the design of feedback controllers, we take an approach inspired by recent advances in feedback optimization of dynamical systems^7–10,21^ and, in particular, we develop a new data-driven optimization approach based on the analytical framework^9^.

#### Formalizing risk tolerance and social objectives

The proposed technical approach builds upon formulating an optimization problem that captures the desired social and economic metrics and incorporates constraints related to risk tolerance.

To this end, let ${h}_{{lim ,i}}$ denote the maximum allowable number of daily hospitalized individuals in the region *i*, and let $\phi _i: [0,1] \rightarrow \mathbb {R}_{\ge 0}$ be a function of the decision variable $u_i$ that models the societal impact induced by the introduction of NPIs in the region *i*^36^; this includes the economic impact of the raised control measures, and/or the societal response to restriction orders. Similarly, let $\psi _i: [0,1] \rightarrow \mathbb {R}_{\ge 0}$ be a function of the number of infectious individuals $\imath _i$ that models the societal losses due to a high number of infections in the region *i*; this includes e.g. the cost of hospitalizations. Mathematically, we assume that $u\mapsto \phi _i(u)$ is a differentiable function^40^ (see Supplementary Information). Since societal losses, in general, do not increase as NPIs are lifted, we also assume that $u\mapsto \phi _i(u)$ is a non-increasing function in its domain. For additional remarks on the cost of NPIs we refer the reader to, e.g.^36,41,42^. To capture the relationship between NPI variables $u = (u_1, u_2 \ldots , n_N)$ and the number of infectious individuals at the endemic equilibrium (i.e. when $t = \infty$), we denote by $u \mapsto \mathscr {F}_i(u)$ the function that maps the instantaneous values of NPIs *u* to the fraction of infections in the *i*-th region at the endemic equilibrium. Hospitalizations can be naturally related to the instantaneous level of NPIs as well as to the current state of the pandemic. To this end, we let $x_i:=(s_i, e_i, \imath _i, h_i, r_i, v_i, d_i)$ denote the joint epidemic state in region *i*, and $x:=(x_1, x_2, \dots , x_N)$ denote the joint epidemic state of the network. We denote by $u \mapsto \mathscr {H}_i(u; x)$ the function that maps the instantaneous level of NPIs *u* into the peak of hospitalizations in region *i*, given the current state *x* of the model.

With these definitions in place, we formulate the problem of optimizing the choice of NPIs while guaranteeing that the number of hospitalizations remains below the pre-specified limit at all times as follows:

3 $$\begin{array}{*{20}l} \min _{\{u_1, \ldots , u_N\}}~~~&\sum _{i = 1}^N \phi _i(u_i) + \psi _i(\mathscr {F}_i(u))&\text {(cost depends on NPI policy and infections in each area)} \nonumber \\ \text {s.t.}~~~&\mathscr {H}_i(u; x) \le {h}_{{lim ,i}}, \,\,\,\,\, i = 1, \ldots , N,&\text {(maximum allowable hospitalizations in area { i})}\nonumber \\&u_i \in [0,1], \quad i = 1, \ldots , N.&\text {(feasible NPI policy for area }i\text {)}. \end{array}$$

Solutions $u^*$ of the above optimization problem, describe a level of NPIs that balances economic and social costs while ensuring that hospitalizations limits are not exceeded in each region. In the following, we develop an optimization-based feedback control method to continuously calibrate the permitted level of social interactions *u* to meet the criteria outlined in the optimization problem (3). We also remark that (3) includes a single-region model as a subcase (see Supplementary Information).

#### Feedback controller design for NPIs

We begin by defining the set of feasible NPIs, as described in the optimization problem (3). For a fixed epidemic state *x*, the feasible region of (3) is given by:

$$\begin{aligned} \mathscr {U}_x := \{ u = (u_1, \dots u_n) \; : \; u_i \in [0,1], \mathscr {H}_i(u; x) - {h}_{{lim ,i}} \le 0, \text { for all } i = 1, \dots N\}. \end{aligned}$$

We note that, because the input-to-peak of hospitalization map $\mathscr {H}(u; x)$ is parametrized by the instantaneous state of the system *x*, the set $\mathscr {U}_x$ is also parametrized by *x*. When the set $\mathscr {U}_x$ is non-convex, we consider a convex approximation $\hat{\mathscr {U}}_x$ as explained in the “Data-driven implementation” section. With this definition, a function $t\mapsto u(t)$ for the NPIs can be obtained as a solution of the following dynamical system:

4 $$\begin{aligned} \dot{u}&= P_{\hat{\mathscr {U}}_x} \big (u - \eta (\nabla \phi (u) + J(u)^\top \nabla \psi (\imath ))\big ) - u, \end{aligned}$$

where $\phi (u) := \sum _{i = 1}^N \phi _i(u_i)$ and $\psi (i) := \sum _{i = 1}^N \psi _i(i_i)$ for brevity, *J*(*u*) is the Jacobian matrix collecting $\{\partial _u \mathscr {F}_i (u)\}_{i=1}^N$ (the notation $\partial _u \mathscr {F}_i (u)$ denotes the gradient of the function $\mathscr {F}_i(\cdot )$), $\eta >0$ is a tunable parameter of the controller, and $P_{\mathscr {U}_x}$ denotes the Euclidean projection operator; namely, given $z \in \mathbb {R}^n$ and a convex set $\mathscr {U} \subseteq \mathbb {R}^n$,

$$\begin{aligned} P_{\mathscr {U}} (z) = \arg \min _{w \in \mathscr {U}} \Vert w-z \Vert . \end{aligned}$$

**Figure 8:**
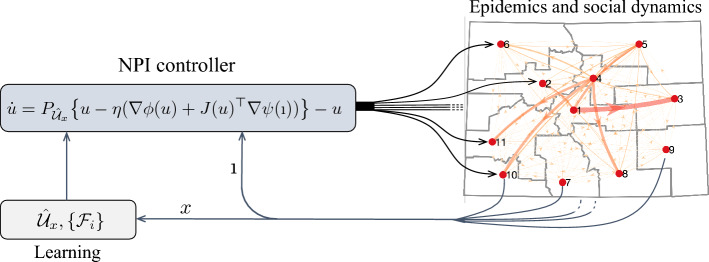
Implementation of the NPI controller. The example refers to the state of Colorado, where each region represents a Local Public Health Agency.

We note that the optimization-based controller (4) leverages two types of feedback: (i) it uses the instantaneous fraction of infectious individuals $\imath$, and (ii) it relies on a projection onto the set $\hat{\mathscr {U}}_x$, which is parametrized by the instantaneous state of the system. For these reasons, the control dynamics (4) describe a *dynamic state-feedback controller* for the NPIs. Critically, the controller relies on the knowledge of the maps $u \mapsto \mathscr {F}_i(u)$ and $u \mapsto \mathscr {H}_i(u; x)$. These maps are estimated from data, as explained in “Data-driven implementation” section. An illustrative example of the implementation of the controllers (4) is provided in Fig. 8.

#### Local implementation

Due to the coupling introduced by the dependence of functions $\mathscr {H}_i$ and $\mathscr {F}_i$ on the (entire) vector of control variables *u*, the implementation of the optimization-based feedback controller (4) critically requires full knowledge of the state, control vector *u*, and of the (gradients of) the cost functions $\phi _1, \dots \phi _N, \psi _1, \dots ,\psi _N$. Therefore, it requires a centralized implementation (for example, at the state level in the example in Fig. 4). When this implementation is not feasible, we consider an approximation of the functions $\mathscr {H}_i$ that accounts only for the effects of the local NPI policies in area *i*, namely, we approximate the value of the peak of hospitalizations $\mathscr {H}_i(u; x)$ by $\tilde{\mathscr {H}}_i(u_i; u_{-i}, x)$, where $u_{-i} = \{u_1, \ldots , u_{i-1}, u_{i+1}, \ldots , u_N \}$ is treated as a constant parameter. By using this approximation, we redefine the set of feasible NPI policies in subregion *i* as:

$$\begin{aligned} \hat{\mathscr {U}}_{x,i} := \{ u \; : \; u_i \in [0,1] \text { and } \tilde{\mathscr {H}}_i(u_i; u_{-i}, x) - {h}_{{lim ,i}} \le 0\}, \end{aligned}$$

where the map $\tilde{\mathscr {H}}_i$ is obtained numerically. By using this approximation, the distributed controller reads as:

5 $$\begin{aligned} \dot{u}_1&= P_{\hat{\mathscr {U}}_{x,1}}[u_1 - \eta (\partial \phi _1(u_1) + \partial _{u_1} \mathscr {F}_1(u) \partial \psi _1(\imath _1))] - u_1, \nonumber \\ \dot{u}_2&= P_{\hat{\mathscr {U}}_{x,2}}[u_2 - \eta (\partial \phi _2(u_2) + \partial _{u_2} \mathscr {F}_2(u) \partial \psi _2(\imath _2))] - u_2, \nonumber \\ \vdots& \vdots \nonumber \\ \dot{u}_N&= P_{\hat{\mathscr {U}}_{x,N}}[u_N - \eta (\partial \phi _N(u_N) + \partial _{u_N} \mathscr {F}_N(u) \partial \psi _N(\imath _N))] - u_N. \end{aligned}$$

Each region *i* can update its NPI policy $u_i$ locally by only relying on the knowledge of: (i) the current fraction of infectious individual $\imath _i$ in region *i*, (ii) the current value of NPI in the network $u_{-i}$, (iii) an estimate of the partial derivative $\partial _{u_i} \mathscr {F}_i(u)$, which can be estimated locally at each region by simulating the model (2), and (iv) the feasible set $\mathscr {U}_{x,i}$. We note that, although the controllers are implemented locally in each region, coordination between regions naturally emerges because of the connectivity in the SEIHRVS model.

#### Data-driven implementation

The maps $\mathscr {H}_i$ and $\mathscr {F}_i$ can be estimated from data using function estimation methods. In particular, to estimate these maps we simulated the dynamics (1) with initial conditions set equal to the instantaneous state of the model, and for different values of the control parameters, chosen in a neighborhood of the current value of *u*. For a set of fixed values for the control parameters, we simulated the dynamics (1) and obtained the values of the peak hospitalizations and the infections at steady state. With these values, we utilized function estimation methods to obtain the maps $\mathscr {H}_i$ and $\mathscr {F}_i$ (see Supplementary Information). We note that the estimated map $\hat{\mathscr {H}}_i$ is required to be convex to build a feasibility set $\hat{\mathscr {U}}_x$ that is convex (which is important in order to have a well-defined projection in our controller)^40,43^.

### Model fitting and data acquisition

#### Model fitting from data

We organized the model-fitting phase into two main stages. First, we fitted the SEIHRVS model by combining model parameters from Ref.^12^ with hospitalization data, and we used a prediction–correction algorithm to minimize the fitting error. The fitted model parameters used in our simulations are reported in Table 1. Second, we used cell-phone usage from SafeGraph (https://docs.safegraph.com) to estimate the interaction matrix of the network. The travel volume from an origin region to destination region on a given date is calculated using the Destination Census Block Groups (CBGs) metric in the social distancing data provided by the Safegraph COVID-19 Data Consortium (https://docs.safegraph.com/docs/social-distancing-metrics). The destination CBGs metric is defined as: The number of devices with a home in [a CBG in origin region] that stopped in [a CBG in destination region] for $> 1$ min [on a given day]. The “home” of the device refers to the most common nighttime location for the device over the prior 6 weeks. The share activity in the region *j* coming from region *i*, used in this analysis, is the activity in *j* from *i* divided by the total activity in *j* across all origins—where “activity in *j* from *i*” is the sum of the destination CBGs metric for all origin CBGs in *i* and all destination CBGs in *j*”. The summation across CBGs does not perform any deduplication, and so the total activity does not represent unique devices on a given day. Instead, it can be interpreted as the total visits to CBGs in the destination region by devices from the origin region, counting a device that stopped for at least a minute in two different CBGs as two visits. This approach has advantages and disadvantages. It may weigh visitors who stay longer and move around during their visit—going to restaurants, parks, and other locales that are not within the same CBG as their hotels—more heavily than visitors whose stay is brief or who limit their movement to a small area. The advantage is that longer stays and more movement tend to carry more risk of COVID-19 transmission, and so it helps us capture the impact of restrictions on travel-induced COVID risk. Additional information on the data is provided in the Supplementary Information.

**Table 1:** Model parameters resulting from the model fitting phase.

Symbol	Value	Description	Source
$\beta$	0.58	Transmission rate	Fitted
$\theta$	0.77	Probability of vaccinating an individual in compartment *s*	^12^
$\delta$	0.02965/365	Daily death/birth rate	^12^
$\sigma$	1/365	1/Duration of natural immunity	^12^
$\eta$	1/730	1/Duration of vaccine immunity	^12^
$\epsilon$	1/4.2	1/Latency period	^12^
$\gamma$	1/9	Rate of recovery	^12^
$\kappa ^{i \rightarrow h}$	0.0143762	Probability of hospitalization after infection	Fitted
$\kappa ^{i \rightarrow d}$	0.00262289	Probability of death after infection	^12^
$\kappa ^{h \rightarrow d}$	0.099204	Probability of death after hospitalization	^12^
$\rho$	1/7.489	1/Hospitalization period	^12^
*y*	15,000–25,000	Vaccination rate	
$\nu$	0.81	Vaccination efficacy	^12^
${N}_{pop }$	5,840,795	State population size, CO, USA	
*s*(0)	1/1.47	Fraction of Susceptible on 03/01/21	^44^
*e*(0)	1/546	Fraction of exposed on 03/01/21	^44^
$\imath (0)$	1/216	Fraction of infectious on 03/01/21	^44^
*h*(0)	1/15936	Fraction of hospitalized on 03/01/21	^44^
*r*(0)	1/4.2136	fraction of recovered on 03/01/21	^44^
*v*(0)	1/13.1	Fraction of vaccinated on 03/01/21	^44^
*u*(0)	0.21	Level of lockdown on 03/01/21	^12^

## Supplementary Information

Supplementary Information.

## Data Availability

Mobility data was obtained from Safegraph, publicly available at the website: https://docs.safegraph.com/docs/social-distancing-metrics. Hospitalization data were obtained from the Colorado Department of Public Health and Environment. Colorado state-wide daily hospitalization was obtained from EMResources, publicly available at https://covid19.colorado.gov/data. Regional-level daily hospitalization census data was obtained from Covid Patient Hospitalization Surveillance, posted publicly at https://github.com/agb85/covid-19/tree/master/Regional%20Models Parameter Values were obtained from previous modeling works^12,13^ and are reported in Table 1 and Supplementary Information.

## Code availability

Software that was custom-developed as part of our methods is available at the repository: https://github.com/gianlucaBi/safe_levels_NPIs.
