# Supplementary material for: Novel use of online optimization in a mathematical model of COVID-19 to guide the relaxation of pandemic mitigation measures
Source: Sci Rep. 2022 Mar 18;12:4731. doi: 10.1038/s41598-022-08389-5 (PMC8932375; doi:10.1038/s41598-022-08389-5)
Supplement: Supplementary file 1 — Supplementary Information. [file 41598_2022_8389_MOESM1_ESM.pdf]

# Supplementary Information

## Planning a Return to Normal after the COVID-19 Pandemic: Identifying Safe Contact Levels via Online Optimization

Gianluca Bianchin[1,*], Emiliano Dall'Anese[1], Jorge I. Poveda[1], David Jacobson[2], Elizabeth Carlton[3], Andrea G. Buchwald[4]

June 29, 2021

[1] Dept. of Electrical, Computer, and Energy Engineering, University of Colorado Boulder
[2] Vanadata, Boulder
[3] Dept. of Environmental and Occupational Health, Colorado School of Public Health, University of Colorado Anschutz
[4] Dept. of Biostatistics and Informatics, Colorado School of Public Health, University of Colorado Anschutz
[*] Corresponding Author

## Supplementary Note 1: Definitions and background material

We provide background notions in analysis and optimization that underpin the proposed technical approach.

We use the following notation. The symbol $\mathbb{R}$ denotes the set of real number, and $\mathbb{R}^n$ denotes the set of $n$-dimensional real vectors. For vectors $u \in \mathbb{R}^n$ and $v \in \mathbb{R}^m$, $(u, v) \in \mathbb{R}^{n+m}$ denotes their concatenation. For a vector $v \in \mathbb{R}^n$, $v^\top$ denotes its transposition; the Euclidean norm of $v$ is given by $\|v\| := \sqrt{v^\top v}$. Consider a function $f : \mathcal{D} \to \mathbb{R}$, where $\mathcal{D} \subseteq \mathbb{R}^n$, and assume that $f(v)$ is differentiable in its domain. We let $\frac{\partial f}{\partial v_i}(v)$ denote the partial derivative of $f$ with respect to the $i$-th component of its argument evaluated at $v$. We denote by $\nabla f(v)$ the gradient of the function $f(\cdot)$, namely $\nabla f(v) = (\frac{\partial f}{\partial v_1}(v), \dots, \frac{\partial f}{\partial v_n}(v))$.

We next provide the definitions of Lipschitz-continuous gradient and convex functions, as well as the definition of convex set and of the projection operator (see [1, 2]).

**Definition 1 (Lipschitz gradient)** *Let $\ell > 0$. A function $f : \mathcal{D} \to \mathbb{R}$, $\mathcal{D} \subseteq \mathbb{R}^n$, has a Lipschitz-continuous gradient over $\mathcal{D}$ if it is differentiable in its domain and it satisfies*

$$\|\nabla f(x) - \nabla f(y)\| \le \ell \|x - y\|, \qquad \forall\, x, y \in \mathcal{D}. \tag{S.1}$$

*In this case, function $f$ is also called $\ell$-smooth.*

**Definition 2 (Convex set)** *A set $\mathcal{U} \subseteq \mathbb{R}^n$ is convex if for any $x, y \in \mathcal{U}$ and any $\lambda \in [0, 1]$, we have $\lambda x + (1 - \lambda) y \in \mathcal{U}$.*

**Definition 3 (Convex function)** *A function $f : \mathcal{D} \to \mathbb{R}$ is convex if $\mathcal{D}$ is a convex set and if for all $x, y \in \mathcal{D}$ and $\lambda \in [0, 1]$, we have*

$$f(\lambda x + (1 - \lambda) y) \le \lambda f(x) + (1 - \lambda) f(y). \tag{S.2}$$

*The function $f$ is strongly convex with constant $\mu > 0$ if $f(x) - \frac{\mu}{2}\|x\|$ is a convex function.*

**Definition 4 (Euclidean Projection)** *Given a convex set $\mathcal{U} \subseteq \mathbb{R}^n$ and a point $z \in \mathbb{R}^n$, the projection of $z$ onto the set $\mathcal{U}$ is*

$$P_{\mathcal{U}}(z) := \arg\min_{x \in \mathcal{U}} \|x - z\|.$$

A prototypical convex optimization problem can be expressed in the form

$$u^* \in \arg\min_{u\in\mathbb{R}^n} \quad f(u), \tag{S.3a}$$

$$\text{subject to:} \quad u \in \mathcal{U}, \tag{S.3b}$$

where $f : \mathbb{R}^n \to \mathbb{R}$ is a convex function and $\mathcal{U} \subseteq \mathbb{R}^n$ is a convex set. The set $\mathcal{U}$ is called feasible set. Problem (S.3) is feasible if the set $\mathcal{U}$ is not empty. We say that $u^*$ is a global minimum of $f$ over $\mathcal{U}$ if $f(u) \geq f(u^*)$ for all $u \in \mathcal{U}$.

An optimization problem in functional form is a problem of the form (S.3) where $\mathcal{U}$ is specified as the intersection of convex sets, namely, $\mathcal{U} := \{g_i(u) \leq 0 \text{ and } h_j(u) = 0, i = 1, \ldots, m, j = 1, \ldots, p\}$, where $g_i : \mathbb{R}^n \to \mathbb{R}$ are convex functions and $h_i : \mathbb{R}^n \to \mathbb{R}$ are affine functions.

**Definition 5 (Stationary point)** *Let $f : \mathcal{D} \to \mathbb{R}$ be differentiable and let $\mathcal{D} \subseteq \mathbb{R}^n$ be a convex set. Then, $x^* \in \mathcal{U}$ is a stationary point of $f$ if $\nabla f(x^*) = 0$.*

**Definition 6 (Stationary point for convex optimization problems)** *Let $f : \mathcal{D} \to \mathbb{R}$ be differentiable and let $\mathcal{D} \subseteq \mathbb{R}^n$ be a closed and convex set. Then, $x^* \in \mathcal{U}$ is a stationary point of the optimization problem* (S.3) *if the condition $\nabla f(x)^\top (x - x^*) \geq 0$ holds for any $x \in \mathcal{U}$.*

A key result in convex optimization [2, Thm. 9.2] states that if $f : \mathcal{D} \to \mathbb{R}$ is a convex and continuously differentiable function and $\mathcal{D} \subseteq \mathbb{R}^n$ is a closed and convex set, then $u^*$ is a global minimum of $f$ over $\mathcal{U}$ if and only if $u^*$ is a stationary point of (S.3). Differently, if $f$ is not convex, then stationary points can be local or global minima, maxima, or saddle points.

By building upon the presented convex optimization framework, we next discuss some properties of the feedback controller (4) utilized in this work. Let $f : \mathcal{D} \to \mathbb{R}$ be a continuously differentiable function and let $\mathcal{U} \subseteq \mathbb{R}^n$ be a closed and convex set. In (4), we we use the following dynamics as a feedback controller for (1):

$$\dot{x} = P_{\mathcal{U}}\big(u - \eta \nabla f(u)\big) - u, \tag{S.4}$$

where $\eta > 0$ is a design parameter. The solutions of the dynamical system (S.4) satisfy the following properties (proven in [3, 4, 5]).

ii) If $f$ has Lipschitz-continuous gradient in its domain, then for each $u_0 \in \mathcal{D}$ there exists a unique continuously differentiable solution $u(t)$ to (S.4) with $u(0) = u_0$ that is defined for all $t \geq 0$.

i) If $u_0 \notin \mathcal{U}$, then $u(t)$ approaches the set $\mathcal{U}$ exponentially-fast. Furthermore, the set $\mathcal{U}$ is forward invariant.

ii) If $f$ is convex, then every stationary point of (S.3) is a Lyapunov stable equilibrium point of (S.4).

iii) If $f$ is strongly convex, then every solution of (S.4) converges to the unique solution of (S.3) exponentially fast.

Finally, we note that if $f$ is not convex, then (S.4) converges to the a stationary point of the problem (S.3); however, only strict local optimizers are asymptotically stable [6].

## Supplementary Note 2: Costs and estimated constraints

As discussed in the main paper, the proposed technical approach builds upon formulating an optimization problem that captures the desired social and economic metrics, and incorporates constraints related to the largest admissible number of hospitalizations. In our work, we select cost functions $\phi_i : [0, 1] \to \mathbb{R}_{\geq 0}$ of the decision variable $u_i$ in region $i$ that model the societal cost induced by the introduction of NPIs in the region, including the economic impact of the raised control measures, and/or the societal response to restriction orders. Mathematically, we require that $u \mapsto \phi_i(u)$ is convex and differentiable function with a

Lipschitz-continuous derivative. Since societal losses do not increase as NPIs are lifted, we also assume that $u \mapsto \phi_i(u)$ is a non-increasing function in its domain. Relevant examples of such a functions include:

$$\text{Example 1: } \phi_i(u_i) = c\left(\frac{1}{u_i} - 1\right), \qquad \text{Example 2: } \phi(u_i) = c(u_i - 1)^2,$$

where $c > 0$ is a free parameter. The Example 1 above was proposed in [7], where the choice of $c$ is made according to the number of employees that are affected by NPIs in the region $i$. Additional functions are discussed in [7], and additional remarks on the cost of NPIs are provided in e.g., [8, 9]. In all the numerical analyses presented in the paper, we used the function Example 2 with $c = 1$.

To capture the relationship between control variables and the number of infectious individuals at the endemic equilibrium (i.e. when $t = \infty$), we denote by $u \mapsto \mathcal{F}_i(u)$ the function that maps the instantaneous values of NPIs $u$ across regions to the fraction of infections in the region $i$ at the endemic equilibrium. For the single-region model (3), a closed-form expression for the this map is available (see Supplementary Note 3); however, for the multi-region model (4), the map $\mathcal{F}_i(u)$ for each region $i = 1, \ldots, N$ is not available in closed form. To estimate each of the maps $\mathcal{F}_i$, several data-based approaches can be used by using the epidemic transmission model as a predictor. In particular, for all the numerical simulations presented in the paper, we simulated the dynamics (4) with initial condition given by instantaneous model state and for different values of the control parameter $\{u^{(1)}, \ldots u^{(p)}\}$. Then, we used use the output of the simulations (precisely, the values of the steady-state infections evaluated at at the trial points $\{u^{(1)}, \ldots u^{(p)}\}$) to construct an approximate map $u \mapsto \mathcal{F}_i(u)$. Alternative functional methods that can be used include Gaussian Processes, Kernel-based Regression, Strongly Convex Regression, or Linear Regression. In the main paper, we approximate the map $\mathcal{F}_i(u)$ using a linear function of the form $\hat{\mathcal{F}}_i(u) = a_i^\top u + b_i$, for $a_i \in \mathbb{R}^N$ and $b \in \mathbb{R}$. In particular, the $p$ test points $\{u^{(1)}, \ldots u^{(p)}\}$ are chosen in a neighborhood of the current vector $u$, and we run the model (4) to obtain the data $\{(u^{(1)}, \mathcal{F}_i(u^{(p)})), \ldots, (\bar{u}^{(p)}, \mathcal{F}_i(u^{(1)}))\}$, where the model is run with initial state given by the current epidemic state $x(0) = x$ and with constant levels of NPIs $\{u^{(1)}, \ldots u^{(p)}\}$. With this data set, define:

$$Y := \begin{bmatrix} u^{(1)\top} & 1 \\ \vdots & \vdots \\ u^{(p)\top} & 1 \end{bmatrix}, \qquad F_{i,x_0} := \begin{bmatrix} \mathcal{F}_i(u^{(1)}) \\ \vdots \\ \mathcal{F}_i(u^{(p)}) \end{bmatrix},$$

where the subscript $x_0$ is used to highlight the dependence on the model state $x$. Then, a linear approximation of $\mathcal{F}_i(u)$ can be found as follows:

$$\hat{\mathcal{F}}_i(u) = a_i^\top u + b_i, \text{ where } \begin{bmatrix} a_i \\ b_i \end{bmatrix} = (Y^\intercal Y)^{-1} Y^\intercal F_{i,x_0}. \tag{S.5}$$

The estimate of the map can be updated to reflect changes in the state $x = \{s_i, e_i, \imath_i, h_i, r_i, v_i\}$; in this case, $\hat{\mathcal{F}}_i(u)$ is a local linear approximant to the true map.

In the main problem formulation, we utilize a function $\imath_i^* \mapsto \psi_i(\imath_i^*)$ that models a cost associated with the infections in region $i$ at the endemic equilibrium; we assume that $\psi_i$ is convex and differentiable function with a Lipschitz-continuous derivative. Examples include:

$$\text{Example 1: } \psi_i(\imath) = k\imath^2, \qquad \text{Example 2: } \psi_i(\imath) = \max\{0, (\imath - \imath^{\text{th}})^2\},$$

where $k \geq 0$ is a parameter and $i^{\text{th}}$ is a given threshold associated with risk tolerance. The parameter $k$ can be used to model the cost incurred by the healthcare system at a given number of infectious individuals (cost of testing, hospitalizations, etc) or economic costs related to possible quarantines and missing work days. Since $\hat{\mathcal{F}}_i(u)$ is linear, we have the the composition of functions $u \mapsto \psi_i(\hat{\mathcal{F}}_i(u))$ is convex and differentiable, and it has a Lipschitz-continuous gradient [2] (under the condition that $\|a_i\| < \infty$ by design). If methods such as Gaussian Processes are utilized to estimate the map $\mathcal{F}_i$, then convexity of $\mathcal{F}_i$ (and, hence, of $\psi_i(\hat{\mathcal{F}}_i(u))$) is not guaranteed. In this case, the convergence and stability properties of gradient flow methods change; in particular, the asymptotic stability of the gradient flow explained in Supplementary Note 1 is a property that holds only for strict local minima.

The technical approach explained in the paper also relies on the map $u \mapsto \mathcal{H}_i(u; x)$ for the region $i$, with $x = \{s_i, e_i, \imath_i, h_i, r_i, v_i\}$, which is a function that maps the instantaneous level of NPIs $u$ (in all the regions)

into the peak of hospitalizations in the region $i$, given the current state of the model. A closed form expression for this map is generally not available; we adopt simulation-based approach similar to the one explained above for the maps $\mathcal{F}_i$, and we utilize a linear approximation of the form $\hat{\mathcal{H}}_i(u;x) = m_i^\top u_i + q_i$ (see Supplementary Fig.2). In this case, the approximate constraint in the problem (3) becomes $m_i^\top u_i + q_i \leq h_{\text{lim},i}$, and it is convex (see Supplementary Note 1).

We conclude by noting that in this work we utilize the Susceptible-Exposed-Infectious-Hospitalized-Recovered-Vaccinated-Susceptible (SEIHRVS) transmission model, explained in the section Methods. However, alternative networked models could be utilized for the simulation-based estimation of the maps (for example, a network version of the recently-proposed model SIDARTHE [10]).

## Supplementary Note 3: Single-region model

In this section, we provide details on the single-region model and on the implementation of the related feedback controller. We recall that the single-region model reads (see (1)):

$$\begin{aligned}
\dot{s} &= -\beta s\imath - \theta\nu y - \delta s + \delta + \sigma r + \eta v,\\
\dot{e} &= -\epsilon e - \delta e + \beta s\imath,\\
\dot{\imath} &= -\gamma\imath - \delta\imath + \epsilon e,\\
\dot{h} &= -\rho h + \kappa^{\imath\to h}\gamma\imath,\\
\dot{r} &= -\sigma r - \delta r - (1-\theta)\nu y + (1-\kappa^{\imath\to h} - \kappa^{\imath\to d})\gamma\imath + (1-\kappa^{h\to d})\rho h,\\
\dot{v} &= -\eta v - \delta v + \nu y,\\
\dot{d} &= \kappa^{\imath\to d}\gamma\imath + \kappa^{h\to d}\rho h,
\end{aligned} \tag{S.6}$$

where we recall that $u \in [0,1]$ specifies the level of permitted social activity. In this case, an exact expression for $\mathcal{F}$ can be derived in closed-form, which reads as:

$$\mathcal{F}(u) = \frac{\delta\left(1 - \frac{(\epsilon+\delta)(\gamma+\delta)}{\epsilon\beta u}\right) - \theta\nu y - \frac{\sigma}{\sigma+\delta}(1-\theta)\nu y + \frac{\eta}{\eta+\delta}\nu y}{\frac{(\epsilon+\delta)(\gamma+\delta)}{\epsilon} - \frac{\sigma}{\sigma+\delta}(1-\kappa^{\imath\to d} - \kappa^{\imath\to h}\kappa^{\imath\to d})}. \tag{S.7}$$

We notice that, when $y=0$ (i.e. the model does not incorporate vaccinations) and $\sigma = 0$ (i.e. there is no loss of immunity), the expression (S.7) simplifies to the well-known input-to-steady state map valid for the SEIR model [11, Section 9.3]:

$$\mathcal{F}(u) = \frac{\epsilon\delta}{(\epsilon+\delta)(\gamma+\delta)}\left(1 - \frac{1}{R_0}\right),$$

where $R_0 := \frac{\epsilon\beta u}{(\epsilon+\delta)(\gamma+\delta)}$ denotes the basic reproduction number.

For this single-region model, the problem of optimizing the severity of NPIs while maintain the number of COVID-19 hospitalizations under a pre-specified threshold (denoted by $h_{\text{lim}}$) can be formulated as follows:

$$\begin{aligned}
\min_u \quad & \phi(u) + \psi(\mathcal{F}(u)) && \text{(loss depends on lockdown policy and infections at steady-state)}\\
\text{s.t.} \quad & \mathcal{H}(u;x) \leq h_{\text{lim}}, && \text{(maximum hospitalizations allowed)}\\
& u \in [0,1], && \text{(u is a feasible NPI)}
\end{aligned} \tag{S.8}$$

where $u \mapsto \mathcal{H}(u;x)$ is a function that maps NPIs into peak hospitalizations. Since $\mathcal{F}(u)$ is increasing in $u$ and $\psi(\imath)$ is convex in its argument, we have that $\psi(\mathcal{F}(u))$ is convex.

The function $\mathcal{H}(u;x)$ can be approximated numerically. In particular, a linear approximation $\hat{\mathcal{H}}(u;x)$ can be found as described in Supplementary Note 3. Alternatively, we also point out that numerical evidences suggest that the map $\mathcal{H}(u;x)$ is monotonically increasing in the variable $u$, for a fixed $x$; in this case, the constraint $\mathcal{H}(u;x) \leq h_{\text{lim}}$ can be converted into $u \leq u_{\text{lim}}$, where $u_{\text{lim}}$ is such that $\mathcal{H}(u_{\text{lim}};x) = h_{\text{lim}}$.

Define the set $\hat{\mathcal{U}}_x := \{u \; : \; u \in [0,1] \text{ and } \hat{\mathcal{H}}(u;x) - h_{\text{lim}} \leq 0\}$ (alternatively, $\hat{\mathcal{U}}_x := [0, \min\{1, u_{\text{lim}}\}]$ if $\mathcal{H}(u;x)$ is monotonically increasing in the variable $u$). The controller proposed in the main paper, in this case, reads as follows:

$$\dot{u} = P_{\hat{\mathcal{U}}_x}[u - \eta(\partial\phi(u) + \partial_u\mathcal{F}(u)\partial\psi(\imath))] - u, \tag{S.9}$$

where $\eta > 0$ is a tunable parameter of the controller. We note that the controller (S.9) leverages two types of feedback from the system:

(i) it uses the instantaneous fraction of infectious individuals $\imath$;

(ii) it relies on a projection onto the set $\hat{\mathcal{U}}_x$, which is parametrized by the instantaneous state of the system.

For these reasons, the control dynamics (S.9) describe a *dynamic state-feedback controller* for the epidemic model (S.6). A block diagram of the controller is illustrated in Supplementary Fig. 1.

We conclude by discussing a special case in which the number of infections at the endemic equilibrium is disregarded from the loss function. In this case, (S.8) can be simplified to optimize the severity of the NPIs while maintaining the hospitalizations under a given limit as follows:

$$\begin{aligned} \min_{u} \quad & \phi(u) && \text{(cost depends on lockdown policy)} \\ \text{s.t.} \quad & \mathcal{H}(u;x) \leq h_{\text{lim}}, && \text{(maximum allowable number of hospitalizations)} \\ & u \in [0,1], && \text{(u is a feasible NPI)}\,. \end{aligned} \tag{S.10}$$

Moreover, we note that, when $\phi(\cdot)$ is a monotonically decreasing function in its domain, an optimal NPI policy that solves (S.10) can also be computed in closed form by letting:

$$\mathcal{H}(u^*;x) = h_{\text{lim}}\,.$$

That is, the restrictions on social interactions are lifted to a level where the forecasted amount of hospitalizations will be precisely at the limit $h_{\text{lim}}$. Although in this case the solution $u^*$ can be found explicitly, one can still utilize the controller in (S.9) to adopt a less aggressive approach for lifting the NPIs. In particular, the controller in (S.9) is simplified in this case as

$$\dot{u} = P_{\hat{\mathcal{U}}_x}[u - \eta\partial\phi(u)] - u, \tag{S.11}$$

where the set $\hat{\mathcal{U}}_x$ is re-estimated on a daily basis based on the current number of infections, vaccinations, and hospitalizations.

## Supplementary Note 4: Stability and convergence of the control method

We next discuss the stability and convergence of the NPI controller when coupled with the dynamics of epidemic transmission.

*Projected gradient flow.* Based on the discussion in Supplementary Note 1, when the set $\mathcal{U}$ is constant over time and the cost function $u \mapsto \phi(u) + \psi(\mathcal{F}(u))$ is convex, continuously differentiable, and has a Lipschitz-continuous gradient over the domain of the function, the projected gradient flow

$$\dot{u} = P_{\mathcal{U}_x}\big(u - \eta(\nabla\phi(u) + J(u)^\top \nabla\psi(\mathcal{F}(u)))\big) - u, \tag{S.12}$$

where $J(u)$ is the Jacobian matrix of $\mathcal{F}$ evaluated at $u$, is globally asymptotically stable [3] (in the sense that every trajectory $u$ converges to the set of minimizers); on the other hand, (S.12) is globally exponentially stable when the cost is strongly convex over the set $\mathcal{U}$ [3], in the sense that every trajectory $u$ converges to the unique minimizer $u^*$ exponentially fast (see also [4, 5] for a proof of these claims)

In our case, the controller relies on the approximate set $\hat{\mathcal{U}}_x$; this set is a polyhedron (it is given by the intersection of finitely many half-spaces) and it is convex. The polyhedron $\hat{\mathcal{U}}_x$ continuously evolves over time since it is parametrized by the state $x(t)$ of the SEIHRVS model. Let $u^*$ denote an optimal solution of the optimization problem

$$\min_{u \in \hat{\mathcal{U}}_x} \phi(u) + \psi(\mathcal{F}(u))$$

and assume that $u^*$ is unique for simplicity of exposition. Under the assumption that the map $t \mapsto \hat{\mathcal{U}}_x$ is locally Lipschitz (and therefore the projection onto moving polyhedra is locally Lipschitz [12]), the solution trajectory $u(t)$ of $\dot{u} = P_{\hat{\mathcal{U}}_x}\big(u - \eta(\nabla\phi(u) + J(u)^\top \nabla\psi(\mathcal{F}(u)))\big) - u$ exists, it is unique, and it can be shown to converge to the optimizer of (S.8) (see [3]).

*Dynamical transmission model.* Consider the single-region Susceptible-Exposed-Infectious-Hospitalized-Recovered-Vaccinated (SEIHRV) model, which corresponds to the dynamical system (2) in the main paper when the loss of immunity is discarded from the model. The SEIHRV dynamical model has an endemic equilibrium that is globally asymptotically stable for any $u$; this can be shown by extending the arguments in [11, Section 9.3] for the single-population Susceptible-Exposed-Infectious-Recovered (SEIR) model.

The networked SEIHRV model has a unique endemic equilibrium. Assuming that the reproduction number is strictly larger than 1 and the interaction graph (modeling mobility) is strongly connected, it can be shown that the endemic equilibrium is globally asymptotically stable [11, Section 9.3].

We conclude this part by noting that the stability of the full dynamical model with the loss of immunity is an open problem; though, we also point out that it is still unclear how to model the loss of immunity of COVID-19 vaccines (the simulations in this work are run for the period of one year, under the assumption that there is no loss of immunity in this time frame).

*Interconnection between controller and dynamics of the epidemic.* The stability of the interconnection between a gradient-flow controller and a dynamical system can be investigated using arguments from singular perturbation theory or small-gain stability [13].

Consider the single-region SEIHRV dynamical model (1) interconnected with the projected gradient flow $\dot{u} = P_{\hat{\mathcal{U}}_x}(u - \eta(\nabla\phi(u) + J(u)^\top \nabla\psi(\imath)) - u$. In this particular case, we have an interconnection between the SEIHRV dynamics, which have an endemic equilibrium that is globally asymptotically stable, and the projected gradient flow. In this setting, it is possible to utilize the procedure explained in [13, 14] to show that there exists a rage of values for the gain $\eta$ of the projected gradient flow that renders the optimizers of the optimization problems input-to-state stable. Similar arguments can be utilized for the interconnection of the multi-region SEIHRV dynamics with the projected gradient flow controller in equation (1) of the main paper.

We conclude by noting that an open problem is to establish stability of the multi-region SEIHRV dynamical model interconnected with the distributed controllers in equation (2) in the main paper; see also Supplementary Fig. 1. The analysis of this interconnection calls for new theoretical results that are not covered by the works of e.g., [3, 6, 13, 14].

## Supplementary Note 5: Data

We organized the model-fitting phase into two main stages. First, we fitted the SEIHRVS model by combining model parameters from [15] with hospitalization data by using a via a prediction-correction algorithm (see table in the main paper). Second, we used cell-phone usage from SafeGraph (`https://docs.safegraph.com/docs/social-distancing-metrics`) to estimate the interaction matrix of the network.

Precisely, model parameters were selected (via a prediction-correction algorithm) to fit hospitalization data for the period from 1/18.21 to 2/28/21. State-wide hospitalization data was obtained from EMResources and regional-level data came from Colorado COVID Patient Hospitalization Surveillance [16, 15]. This time-interval was selected due to a steady decline in hospitalizations observed over this period, thus overcoming the need to account for varying transmission rates during model-fitting. Validation was carried out by comparing the evolution of the parametrized model with hospitalization data, and by assessing performance through a mean squared error. For the parametrized model, the mean squared error associated with hospitalizations and infections was less than 10% over the entire dataset. The model was initialized on 1/18/21 by using infection data from [17]. The state of the pandemic on 03/01/21 was computed by initializing the model on 1/18/21 and by simulating the evolution of the epidemic with constant vaccination rate of $y = 12,000$ vax/day and vaccination efficacy $\nu = 0.9$.

Cell-phone data usage was used to estimate the mobility matrix. Data was obtained from SafeGraph, in the form of counts of cell phone accounts that normally reside in county $i$ and traveled to county $j$ on a specific date, have been averaged over the time interval 03/01/20–12/31/2020.

All computational analyses and the fitting of data were performed using MATLAB and the corresponding optimization toolbox. Our Montecarlo simulations suggest that the outcomes of our simulations are robust to variations of the parameter, confirming their validity to describe reality. Note that the model describing the epidemic spread is highly nonlinear and therefore potentially sensitive to parameter perturbations.

## Supplementary Figures and Tables

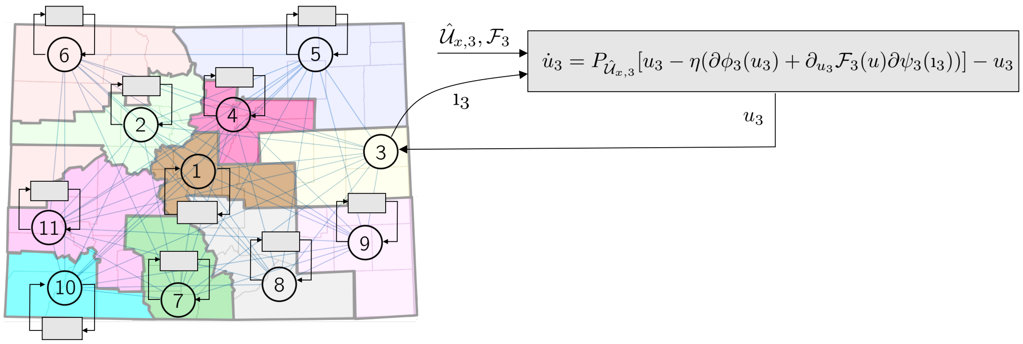


Supplementary Fig. 1: *Local implementation of the NPI controller in each region. Each local controller relies on the current fraction of infectious individuals in the region of interest and the set of feasible NPI strategies $\hat{\mathcal{U}}_{x,i}$; the latter is calculated based on the limit of hospitalizations and a projection of the progression of the infections using the SEIHRVS model. With these inputs, the controller yields a new value for $u_i$ based on the economic and social metrics defined by the functions $\phi_i$ and $\psi_i$.*

Supplementary Fig. 2: *Map $\mathcal{H}_i(u_i, x)$ estimated via simulations, and approximate map computed according to* (S.5) *in a neighborhood of the current epidemic state.*

## Supplementary References


[1] S. Boyd and L. Vandenberghe, *Convex optimization.* Cambridge university press, 2004.

[2] A. Beck, *Introduction to nonlinear optimization: Theory, algorithms, and applications with MATLAB.* SIAM, 2014.

[3] G. Bianchin, J. Cortés, J. I. Poveda, and E. Dall'Anese, "Time-varying optimization of LTI systems via projected primal-dual gradient flows," *arXiv preprint*, Jan. 2021, arXiv:2101.01799.

[4] Y. Xia and J. Wang, "On the stability of globally projected dynamical systems," *Journal of Optimization Theory and Applications*, vol. 106, no. 1, pp. 129–150, 2000.

[5] X.-B. Gao, "Exponential stability of globally projected dynamic systems," *IEEE Trans. on Neural Networks*, vol. 14, no. 2, pp. 426–431, 2003.

[6] A. Hauswirth, S. Bolognani, G. Hug, and F. Dörfler, "Timescale separation in autonomous optimization," *IEEE Transactions on Automatic Control*, 2020, (In press).

[7] Q. Ma, Y.-Y. Liu, and A. Olshevsky, "Optimal lockdown for pandemic stabilization," *arXiv preprint*, 2020, arXiv:2010.12923.

[8] S. Chen *et al.*, "Tracking the economic impact of COVID-19 and mitigation policies in Europe and the United States," *IMF Working Papers*, 2020, working Paper No. 20/125.

[9] A. Ugarov, "Inclusive costs of NPI measures for COVID-19 pandemic: three approaches," *medRxiv*, 2020, medRxiv:2020.03.26.20044552.

[10] G. Giordano, F. Blanchini, R. Bruno, P. Colaneri, A. D. Filippo, A. D. Matteo, and M. Colaneri, "Modelling the COVID-19 epidemic and implementation of population-wide interventions in Italy," *Nature Medicine*, vol. 26, no. 6, pp. 855–860, 2020.

[11] M. Mesbahi and M. Egerstedt, *Graph theoretic methods in multiagent networks.* Princeton University Press, 2010.

[12] E. M. Bednarczuk and K. E. Rutkowski, "On lipschitz continuity of projections onto polyhedral moving sets," *Applied Mathematics & Optimization*, pp. 1–29, 2020.

[13] H. K. Khalil and J. W. Grizzle, *Nonlinear systems*, 3rd ed. Prentice hall Upper Saddle River, NJ, 2002.

[14] A. R. Teel, L. Moreau, and D. Nesic, "A unified framework for input-to-state stability in systems with two time scales," *IEEE Transactions on Automatic Control*, vol. 48, no. 9, pp. 1526–1544, 2003.

[15] A. G. Buchwald *et al.*, "Colorado COVID–19 mathematical model documentation," https://agb85.github.io/covid-19/SEIR%20Documentation.pdf, 2021, [Online; accessed 11-Apr-2021].

[16] ——, "COVID-19 regional models for colorado," https://agb85.github.io/covid-19/Regional%20Report.html, 2021, [Online; accessed 9-Apr-2021].

[17] ——, "The current state of COVID-19 in Colorado 01/20/21," https://coloradosph.cuanschutz.edu/docs/librariesprovider151/default-document-library, 2021, [Online; accessed 18-March-2021].
